# Long term follow-up of humoral and cellular response to mRNA-based vaccines for SARS-CoV-2 in patients with active multiple myeloma

**DOI:** 10.3389/fonc.2023.1208741

**Published:** 2023-05-25

**Authors:** Katia Mancuso, Elena Zamagni, Vincenza Solli, Liliana Gabrielli, Marta Leone, Lucia Pantani, Serena Rocchi, Ilaria Rizzello, Paola Tacchetti, Stefano Ghibellini, Emanuele Favero, Margherita Ursi, Marco Talarico, Simona Barbato, Ajsi Kanapari, Flavia Bigi, Michele Puppi, Carolina Terragna, Enrica Borsi, Marina Martello, Andrea Poletti, Alessandra Scatà, Giuliana Nepoti, Barbara Ruffini, Tiziana Lazzarotto, Michele Cavo

**Affiliations:** ^1^ IRCCS Azienda Ospedaliero-Universitaria di Bologna, Istituto di Ematologia “Seràgnoli”, Bologna, Italy; ^2^ Dipartimento di Scienze Mediche e Chirurgiche, Università di Bologna, Bologna, Italy; ^3^ Microbiology Unit, IRCCS Azienda Ospedaliero-Universitaria di Bologna, Bologna, Italy; ^4^ Microbiology, Department of Medical and Surgical Sciences, University of Bologna, Bologna, Italy

**Keywords:** multiple myeloma, SARS-CoV-2, mRNA-vaccines, immunogenicity, humoral response, cellular response

## Abstract

Long-term kinetics of antibody (Ab) and cell-mediated immune (CMI) response to full anti-SARS-CoV-2 vaccine schedule and booster doses in Multiple Myeloma (MM) patients remain unclear. We prospectively evaluated Ab and CMI response to mRNA vaccines in 103 SARS-CoV-2-naïve MM patients (median age 66, 1 median prior line of therapy) and 63 health-workers. Anti-S-RBD IgG (Elecsys^®^assay) were measured before vaccination and after 1 (T1), 3 (T3), 6 (T6), 9 (T9) and 12 (T12) months from second dose (D2) and 1 month after the introduction of the booster dose (T1D3). CMI response (IGRA test) was evaluated at T3 and T12. Fully vaccinated MM patients displayed high seropositivity rate (88.2%), but low CMI response (36.2%). At T6 the median serological titer was halved (p=0.0391) in MM patients and 35% reduced (p=0.0026) in controls. D3 (94 patients) increased the seroconversion rate to 99% in MM patients and the median IgG titer in both groups (up to 2500 U/mL), maintained at T12. 47% of MM patients displayed a positive CMI at T12 and double-negativity for humoral and CMI (9.6% at T3) decreased to 1%. Anti-S-RBD IgG level ≥346 U/mL showed 20-times higher probability of positive CMI response (OR 20.6, p<0.0001). Hematological response ≥CR and ongoing lenalidomide maintenance enhanced response to vaccination, hindered by proteasome inhibitors/anti-CD38 monoclonal antibodies. In conclusion, MM elicited excellent humoral, but insufficient cellular responses to anti-SARS-CoV-2 mRNA vaccines. Third dose improved immunogenicity renewal, even when undetectable after D2. Hematological response and ongoing treatment at vaccination were the main predictive factors of vaccine immunogenicity, emphasizing the role of vaccine response assessment to identify patients requiring salvage approaches.

## 1 Introduction

Bacterial and viral infections secondary to disease-related immune dysfunction and therapy-related immunosuppression are a major cause of morbidity and mortality in Multiple Myeloma (MM) (1). Accordingly, an increased risk of severe coronavirus disease 2019 (COVID-19) was reported for MM patients (2, 3), with a case fatality rate of 33% (4).

To decrease the spread of the disease and the severity of illness, vaccination against severe acute respiratory syndrome coronavirus 2 (SARS-CoV-2) is strongly recommended for patients with active and smoldering MM (SMM), as well as for those with monoclonal gammopathy of undetermined significance (1, 5, 6). Anti SARS-CoV-2 vaccines initially authorized for use in the European Union employ either the mRNA technology (e.g. BNT162b2 by Pfizer BioNTech, and mRNA-1273 by Moderna) or inactivated adenoviruses as vector (e.g. ChAdOx1-s by AstraZeneca, and Ad26.COV2.S by Janssen). Later on, two additional vaccines using either a recombinant spike protein (e.g. NVX-CoV2373 by Novavax) or an inactivated virus (e.g. VLA2001 by Valneva) granted EMA approval. All these vaccines showed effectiveness in preventing COVID-19 in up to 95% healthy adults (7, 8). A number of subsequent studies in patients with active MM or precursor conditions consistently reported an impaired humoral response to two doses of SARS-CoV-2 vaccination, although with variable failure rates of seroconversion, likely reflecting differences between studies and patients characteristics (9–16). Indeed, several host-, disease-, and treatment-related factors influence the lower immunogenicity rate in MM (11, 13, 14, 16). To enhance or restore the protection against COVID-19, which may decrease over time, a third primary dose (i.e. the final dose of the primary mRNA-based vaccination schedule), and eventually subsequent booster doses, were subsequently recommended for immunocompromised people, including MM patients. However, many of these studies included short-term analyses of immune response kinetics and did not evaluate the immunogenicity of the third dose, which still remains poorly explored in MM patients (17–20). Additionally, although T‐cell response plays an important role in vaccine protection against viral variants and severe COVID-19 disease (21), cellular immunity has not been widely explored. Conflicting results have been reported on the proportion of MM patients who elicited T‐cell responses following the first two doses (16, 22–25), and even fewer data have been published after the third dose of vaccine (17, 18).

To address several of these issues, we designed an observational, single-center, prospective cohort study aimed at evaluating the rates and long-term kinetics of both humoral and cell-mediated immune (CMI) response to three doses of COVID-19 mRNA vaccination for MM patients in a real-world setting. Additional end-points of the study included the correlation between immunogenicity and patients’ immune, disease and response status before vaccination, exposure to prior treatments, and type of vaccine.

## 2 Materials and methods

### 2.1 Study design

The study included patients aged ≥18 years with a confirmed diagnosis of active MM in any treatment line and who had subsequent access to our ambulatory care services (26). Patients initially received at least two consecutive doses (i.e. D1 and D2) of BNT162b2 or mRNA1273 vaccines, three or four weeks apart respectively, according to international recommendations. After EMA approval of the booster dose (D3), the initial study design was amended to include evaluation of third dose immune efficacy. Patients with a diagnosis of SMM, or prior SARS-CoV-2 infection, or with seropositive test prior to vaccination were excluded from the analysis.

Sixty-three health-care workers receiving a full immunization schedule of mRNA vaccine served as controls.

To exclude prior SARS-Cov-2 infection, serum neutralizing IgG antibodies to the receptor binding domain (RBD) of the S1 subunit of SARS-CoV-2 spike (S) protein (anti S-RBD IgG) and anti-nucleocapsid (N) antibodies titers were evaluated within 10 days before the first dose. Anti S-RBD IgG levels were subsequently assessed by employing the same platform to longitudinally monitor the long-term kinetics of humoral response to vaccination at definite timepoints, which included the first (T1), third (T3), sixth (T6), ninth (T9), and twelfth (T12) month following the second vaccine dose (D2) and 1 month after the third dose (T1D3), when introduced.

Cellular immunogenicity was assessed simultaneously with the humoral response at T3 and T12.

Any discontinuation or modification of anti-MM treatment regimens were planned according to the recommendations of the International Myeloma Society (IMS) (5).

MM participants filled in a survey reporting any adverse event and side effect following each vaccine dose, graded according to the Common Terminology Criteria for Adverse Events ver. 5.0. Breakthrough infections were graded according to the WHO severity criteria for COVID-19 (WHO/2019-nCoV/Clinical/2022.2).

The study was approved by the Ethics Committee of the National Institute for Infectious Diseases Lazzaro Spallanzani in Rome and by the local Ethics Committee and was conducted in accordance with the International Conference on Harmonisation Guidelines on Good Clinical Practice and the principles of the Declaration of Helsinki. All participants signed an informed consent form prior to their inclusion in the study.

### 2.2 Immune response laboratory evaluations

Total levels of anti-SARS-CoV-2 spike antigen antibodies (predominantly IgG) (S-RBD) were measured using the FDA approved Elecsys^®^ Anti-SARS-CoV-2 ECLIA assay Roche Diagnostics EG on the cobas e 801 analyzer (27). Manufacturer recommendations for evaluation of results were implemented by including 3 different cut-off values to accurately stratify patients into the following subgroups: negative (<0.8 U/mL); inconclusive (0.8 to <5 U/mL); and positive (≥5 U/mL).

Results for anti-N antibodies were expressed as “present” or “absent”, on the basis of a cut-off index (COI) ≥1.0 and <1.0, respectively.

SARS-CoV-2 CMI response was assessed by the IGRA (Interferon-Gamma Release Assays) QuantiFERON Human IFN-gamma SARS-CoV-2, Qiagen^®^ (QFN SARS) assay, an *in vitro* diagnostic test designed for the qualitative detection of interferon-γ (IFN-γ), produced by CD4+ and CD8+ T cells, in response to stimulation by a SARS-CoV-2 peptide cocktail in heparinized whole blood. Details about the performance and interpretation of the IGRA assay are provided in the supplementary materials and **Table 1S**.

### 2.3 Statistical analysis

Univariate analysis was performed to describe the variables, with continuous variables reported as median and inter-quantile range (IRQ) and qualitative variables reported as absolute frequency and percentages. Chi-2 test was used for assessing the presence of significant association between qualitative variables; when necessary, univariate odds ratio were computed as well, to quantify the size and direction of the statistical relation and relative p-values. The non-parametrical Kruskal Wallis test was applied to assess median differences between groups, with a significance level of 0.05. Multivariate analysis was performed by GLM linear models for anti S-RBD IgG and by logistical models for CMI response; estimated coefficients and odds ratios were presented by forest plots, with confidence intervals (CIs) at 95%.

The anti S-RBD IgG cut-off value was identified by means of ROC curve, with the aim to naturally dichotomize a continuous biomarker into high and low levels.

All data analyses were performed by R-studio (1.4.3).

## 3 Results

### 3.1 Study population

One hundred and three MM patients, who received at least two doses of SARS-CoV-2 mRNA vaccines between April and June 2021, were enrolled into the study. Due to the presence of anti-N antibodies at the baseline evaluation, one patient was excluded from the analysis. Among the remaining 102 SARS CoV-2-naïve MM patients, BNT162b2 and mRNA1273 vaccines were given to 44 (43.1%) and 58 (56.9%) patients. Of these, 94 patients were subsequently treated with the third dose (BNT162b2 in 75.5% and mRNA1273 in 24.5% of them). The main characteristics, disease status and prior treatments at study entry of the overall patient population are summarized in **Table 1**. The median age was 66 (range 47-83) years, and the median number of previous lines of therapy was 1 (range 1-11); most of the patients were actively receiving anti-MM therapy at the time of vaccination, while 7 (6.9%) patients were treatment-free. Forty-five (44.1%) patients had relapsed/refractory MM (RRMM) while the remaining were undergoing first-line therapy. Among 73 (71.6%) transplant-eligible patients, 63 patients had actually received autologous stem-cell transplantation (ASCT) within a median of 32 (16-68) months before vaccination. Responses to the last anti-MM therapy before vaccination in the overall patient population were as follows: very good partial response (VGPR) or higher in 71 (69.6%), partial response (PR) in 17 (16.7%), stable disease (SD) in 5 (4.9%) and progressive disease (PD) in 9 (8.8%) patients. Immunoparesis, as defined by ≥1 uninvolved immunoglobulin (Ig) class concentration below the lower limit, was observed in 83 (88.3%) patients, of whom 64 (68.1%) had a reduction of both Ig classes; lymphopenia (≤1.0 lymphocytes x 10^9^/L) and neutropenia (≤1.0 granulocytes x 10^9^/L) were observed in 3.9% and 21.6% of patients, respectively. The schedule of on-going anti-MM treatments before vaccination was modified in 36 patients, and mainly consisted in transient dexamethasone and/or daratumumab holding. Thirty-three patients who were receiving lenalidomide maintenance continued treatment without any discontinuation. Sixty-three health-care workers (median age: 63 [range 50-67] years) who received mRNA vaccination during the same period represented the control cohort of the study, regarding humoral response only.

**Table 1:** Patients and treatment characteristics at enrolment.

Variable	MM population
Patients, N	102
Median age at vaccination (range), years	66 (47-83)
Sex (female/male), N	48/54
Immunoglobulin isotype, N (%)	
IgG	54 (52.9)
IgA	20 (19.6)
IgD	2 (2)
BJ	9 (8.8)
Light chain κ or λ	17 (16.7)
ISS, N (%) [n=89 evaluable]	
I	41 (46)
II	28 (31.5)
III	20 (22.5)
FISH cytogenetics, N (%) [n=79 evaluable]	
Standard risk	62 (78.5)
High-risk^a^	17 (21.5)
Absolute lymphocyte count, N (%)	
Lymphopenia ≤1.0 x 10 (9)/L	4 (3.9)
Lymphopenia ≤0.5 x 10 (9)/L	0
Absolute neutrophil granulocytes count, N (%)	
Neutropenia ≤1.0 x 10 (9)/L	22 (21.6)
Neutropenia ≤0.5 x 10 (9)/L	5 (4.9)
Immunoglobulins, median (IQR) mg/dl [n=94 evaluable]	
IgG	802 (338-1067.8)
IgA	174 (17-134.3)
IgM	30 (20-39)
Immunoparesis^b^, N (%) [n=94 evaluable]	
≥1 immunoglobulin class	83 (88.3)
2 immunoglobulin classes	64 (68.1)
Time since MM diagnosis, median (range) months	38.5 (0-281)
Active treatment at time of vaccination, N (%)	95 (93.1)
Lines of therapy received, median (range)	1 (1-11)
1, N (%)	57 (55.9)
2, N (%)	30 (29.4)
≥3, N (%)	15 (14.7)
Treatment regimen at vaccination, N (%)	
IMiDs	74 (77.9)
PIs	32 (33.7)
Anti-CD38 MoAbs	33 (34.7)
IMiDs + PIs	14 (14.7)
Lenalidomide maintenance	33 (34.7)
Prior ASCT, N (%)	63 (61.8)
≤12 months	14 (22.2)
>12 months	49 (77.8)
Time since ASCT, median (IQR) months	32 (16-68)
MM treatment response at vaccination, N (%)	
≥CR	50 (49)
VGPR	21 (20.6)
PR	17 (16.7)
SD	5 (4.9)
PD	9 (8.8)
Vaccine type D2, N (%)	
mRNA-1273	58 (56.9)
BNT162b2	44 (43.1)
Vaccine type D3, N (%) [n=99 evaluable]	94 (94.9)
mRNA-1273	71 (75.5)
BNT162b2	23 (24.5)
Time between D2 and D3, median (IQR) months	6 (5-7)

^a^High-risk defined by the presence of one or more of the following abnormalities: del(17p), t(4;14), or t(14;16).

^b^Immunoparesis defined by uninvolved immunoglobulin class concentration below the lower limit.

MM, multiple myeloma; N, number; %, percentage; ISS, international staging system; FISH, fluorescence in situ hybridization; IMiDs, immunomodulatory drugs; PIs, proteasome inhibitors; ASCT, autologous stem cell transplantation; IQR, interquartile range; CR, complete response; VGPR, very good partial response; PR, partial response; SD, stable disease; PD, progressive disease; D2, second vaccine dose; D3, third vaccine dose.

### 3.2 Humoral immune response

At a median of 30 (IQR 28-32) days following D2, a seropositive response, as previously defined, was found in 90/102 (88.2%) MM patients vs a 100% immunogenicity rate in healthy controls (p<0.0001). Median levels of anti S-RBD IgG in these subgroups were 481 (range 0.4-2500) U/mL vs 835 (range 118-2500) U/mL, respectively (p=0.0031). At a median of 3 (IQR 2-4) months after vaccination (T3), serological response was evaluated in 100 out of 102 patients, the remaining 2 having died due to MM. The rates of seroconversion in both study cohorts were almost superimposable to those observed at T1 (90% for MM patients); comparisons between MM patients and healthy controls significantly favoured these latter in terms of higher seroconversion rate and median antibody levels (**Table 2**). At a median of 5 (IQR 4-7) months following D2 (T6) the seroconversion rate remained high in both MM patients (n=92/99, 93%) and controls (n=63, 100%). Notably, two patients (without evidence of COVID-19 infection and treated immediately after D2 with ASCT and CAR T-cell therapy, respectively) showed a late seroconversion, whereas none of the seropositive patients became seronegative. However, the median titers of antibodies were significantly lower than at T1, with a reduction of about 50% in MM patients (240 vs 481 U/mL, respectively, p=0.0391), and a 35% decline in the control group (539 vs 835 U/mL, p=0.0026).

**Table 2:** Humoral response to sars-cov-2 mRNA vaccines in MM patients and healthy controls.

Timepoints (months) median (range)	Population N evaluable	Seroconversion^a^ N (%)		Anti S-RBD IgG level median (range), U/mL	
T1 1 (1-2)	MM 102	90 (88%)	p<0.0001	481 (0.4-2500)	p=0.0031
	Controls 63	63 (100%)		835 (118-2500)	
T3 3 (2-4)	MM 100	90 (90%)	p<0.0001	308 (0.4-2500)	p<0.0001
	Controls 63	63 (100%)		840 (131-2500)	
T6 5 (4-7)	MM 99	92 (93%)	p<0.0001	240 (0.4-2500)	p=0.0062
	Controls 63	63 (100%)		539 (92-2500)	
T1D3^b^ 1 (1-3)	MM 94	88 (94%)	/	2500 (0.4-2500)	/
T9 8 (7-10)	MM 96	93 (97%)	p=0.0013	2500 (0.4-2500)	p<0.0001
	Controls 49	49 (100%)		411 (45-2500)	
T12 11 (9-12)	MM 93	92 (99%)	p=0.1282	2500 (0.4-2500)	p=0.0007
	Controls 21	21 (100%)		2500 (445-2500)	

IgG anti S-RBD were measured at 1 (T1), 3 (T3), 6 (T6), 9 (T9) and 12 (T12) months following the second dose (D2) of vaccination.

^a^Seroconversion was defined by anti S-RBD IgG level ≥5 U/mL.

^b^94 patients received the third dose (D3) of mRNA vaccine at a median of 6 (IQR 5-7) months after D2. An additional assessment of anti S-RBD IgG was planned one month after D3 (T1D3).

S-RBD, receptor binding domain (RBD) of the S1 subunit of SARS-CoV-2 spike (S) protein; Ig, immunoglobulins; MM, multiple myeloma; N, number; %, percentage; p, pvalue.

D3 vaccines were administered at a median of 6 (IQR 5-7) months after D2. The kinetics of humoral response after D3 is summarized in **Table 2**. At a median of 5 (IQR 4-6) months after the booster dose (corresponding to T12), the seroconversion rate in the MM subgroup was as high as 99%, a value superimposable to the 100% seen in healthy controls (p=0.1282). In addition, the median anti S-RBD IgG titer reached the maximum measurable level (2500 U/mL) after D3 in both groups, resulting in at least a 10-fold increase in MM patients and without a significant drop at T12 (p=0.9999).

Univariate analyses of variables potentially influencing humoral response at each of the prespecified time-points (**Table 3**) revealed a relationship between antibody titers and having received the first two doses of mRNA-1273 vaccine, attainment of at least complete response (CR), and being on treatment with lenalidomide maintenance at the time of vaccination. In addition, patients who underwent ASCT showed a median anti S-RBD IgG level significantly higher than the others. Conversely, immunoparesis with involvement of both Ig classes, and prior anti-MM therapies including PIs (proteasome inhibitors) or anti-CD38 monoclonal antibodies (MoAbs), were associated with an impaired humoral response at all time-points (**Table 3**). Notably, patients receiving PIs or anti-CD38 MoAbs-containing therapies displayed a further marked reduction of the median antibody titer (597 U/mL and 1567 U/mL, respectively) at T12 compared with the other patients (2500 U/mL) (p=0.0061 and p=0.0172 for each comparison, respectively), as well as patients with immunoparesis involving both Ig classes (2012 vs 2500 U/mL for the others, p=0.0290).

**Table 3:** Univariate analysis of variables influencing humoral response at the different time-points.

	T1		T3		T6		T1D3		T9		T12	
	Median anti S-RBD IgG, mg/dl (range)											
Variables	YES	NO	YES	NO	YES	NO	YES	NO	YES	NO	YES	NO
PMN ≤1.0 x 10 (9)/L p-value	629 (0.4 - 2500)	103 (0.4 - 2500)	95 (0.4 - 2500)	350 (0.4 - 2500)	53 (0.4 - 2500)	380 (0.4 - 2500)	1889 (0.4 - 2500)	2500 (0.4 - 2500)	1079 (0.4 - 2500)	2500 (0.4 - 2500)	709 (29 - 2500)	2500 (0.4 - 2500)
	**0.011**		**0.083**		**0.085**		**0.003**		**0.005**		**0.012**	
Immunoparesis^a^ p-value	207 (0.4 - 2500)	1921 (59 – 2500)	173 (0.4 - 2500)	972 (30 - 2500)	130 (0.4 - 2500)	643 (20 - 2500)	2500 (0.4 - 2500)	2500 (91 – 2500)	2379 (0.4 - 2500)	2500 (5 – 2500)	2012 (10 - 2500)	2500 (29 - 2500)
	**<0.001**		**<0.001**		**0.003**		**0.032**		**0.227**		**0.029**	
mRNA–1273^b^ (D1+D2) p-value	1921 (0.4 - 2500)	177 (0.4 - 2500)	583 (0.4 - 2500)	94 (0.4 - 2500)	495 (0.4 - 2500)	80 (0.4 - 2500)	2500 (0.4 - 2500)	2500 (3 – 2500)	2500 (0.4 - 2500)	1784 (0.4 - 2500)	2500 (0.4 - 2500)	1434 (29 - 2500)
	**<0.001**		**0.001**		**0.001**		**0.077**		**0.015**		**0.018**	
mRNA–1273 (D3) p-value	- -	- -	- -	- -	- -	- -	2500 (0.4 - 2500)	2135 (3 – 2500)	2500 (0.4 - 2500)	1193 (5 – 2500)	2500 (0.4 - 2500)	865 (32 – 2500)
	–		–		–		**0.019**		**0.01**		**0.001**	
Prior lines >1 p-value	184 (0.4 - 2500)	1077 (0.4 - 2500)	94 (0.4 - 2500)	532 (0.4 - 2500)	113 (0.4 – 2500)	474 (4 - 2500)	2500 (0.4– 2500)	2500 (20 - 2500)	1919 (0.4 - 2500)	2500 (0.4 - 2500)	1883 (0.4 - 2500)	2500 (29 – 2500)
	**<0.001**		**0.001**		**0.003**		**0.005**		**0.072**		**0.066**	
PIs regimen p-value	156 (0.4 - 2500)	892 (0.4 - 2500)	95 (0.4 - 2500)	518 (0.4 - 2500)	74 (1 – 2500)	480 (0.4 - 2500)	2500 (7 – 2500)	2500 (0.4 - 2500)	1394 (0.4 - 2500)	2500 (0.4 - 2500)	597 (29 - 2500)	2500 (0.4 - 2500)
	**0.001**		**0.001**		**0.004**		**0.442**		**0.021**		**0.006**	
IMiDs + PIs regimen p-value	231	567	146	348	80	400	1963	2500	758	2500	440	2500
	(0.4 - 2500)	(0.4 - 2500)	(3 – 2500)	(0.4 - 2500)	(12 - 2500)	(0.4 - 2500)	(7 – 2500)	(0.4 - 2500)	(0.4 - 2500)	(0.4 - 2500)	(29 - 2500)	(0.4 - 2500)
	**0.223**		**0.188**		**0.07**		**0.087**		**0.008**		**0.014**	
Anti-CD38 MoAbs regimen p-value	175	1042	115	382	102	468	2500	2500	1850	2500	1567	2500
	(0.4 - 2500)	(0.4 - 2500)	(0.4 - 2500)	(0.4 - 2500)	(0.4 – 1729)	(0.4 - 2500)	(0.4 - 2500)	(0.4 - 2500)	(19 – 2500)	(0.4 - 2500)	(10 – 2500)	(0.4 - 2500)
	**<0.001**		**0.012**		**0.009**		**0.404**		**0.17**		**0.017**	
Lenalidomide maintenance p-value	2473 (179 – 2500)	205 (0.4 - 2500)	1493 (18.4 – 2500)	148 (0.4 - 2500)	1169 (18 – 2500)	119 (0.4 - 2500)	2500 (532 – 2500)	2500 (0.4 - 2500)	2500 (399 – 2500)	1784 (0.4 - 2500)	2500 (679 – 2500)	1188 (0.4 - 2500)
	**<0.001**		**<0.001**		**<0.001**		**<0.001**		**<0.001**		**<0.001**	
Prior ASCT p-value	1188 (0.4 - 2500)	205 (0.4 - 2500)	658 (0.4 - 2500)	119 (0.4 - 2500)	595 (0.4 - 2500)	59 (0.5 – 2500)	2500 (46 – 2500)	1860 (0.4 - 2500)	2500 (19 – 2500)	1050 (0.4 - 2500)	2500 (10 - 2500)	748 (0.4 - 2500)
	**<0.001**		**<0.001**		**<0.001**		**<0.001**		**<0.001**		**<0.001**	
≥CR p-value	1296 (0.4 - 2500)	218 (0.4 - 2500)	628 (0.4 - 2500)	182 (0.4 - 2500)	489 (0.4 - 2500)	59 (0.4 - 2500)	2500 (0.4 - 2500)	2188 (0.4 - 2500)	2500 (0.4 - 2500)	1380 (0.4 - 2500)	2500 (29 - 2500)	985 (0.4 - 2500)
	**<0.001**		**0.002**		**0.001**		**0.002**		**0.001**		**0.011**	
≥VGPR p-value	903 (0.4 - 2500)	152 (0.4 - 2500)	473 (0.4 - 2500)	91 (0.4 - 2500)	404 (0.4 - 2500)	52 (0.4 – 2401)	2500 (0.4 - 2500)	1048 (0.4 - 2500)	2500 (0.4 - 2500)	1345 (0.4 - 2500)	2500 (29 - 2500)	985 (0.4 - 2500)
	**<0.001**		**0.007**		**0.002**		**0.002**		**0.018**		**0.094**	
≥PR p-value	628 (0.4 - 2500)	206 (0.4 - 2500)	350 (0.4 - 2500)	91 (0.4 - 2500)	272 (0.4 - 2500)	93 (0.4 – 1399)	2500 (0.4 - 2500)	715 (0.4 - 2500)	2500 (0.4 - 2500)	1380 (0.4 - 2500)	2500 (10 - 2500)	1705 (0.4 - 2500)
	**0.122**		**0.055**		**0.097**		**0.012**		**0.266**		**0.566**	
≤SD p-value	206 (0.4 - 2500)	628 (0.4 - 2500)	91 (0.4 - 2500)	350 (0.4 - 2500)	93 (0.4 – 1399)	272 (0.4 - 2500)	715 (0.4 - 2500)	2500 (0.4 - 2500)	1380 (0.4 - 2500)	2500 (0.4 - 2500)	1705 (0.4 - 2500)	2500 (10 - 2500)
	**0.122**		**0.055**		**0.097**		**0.012**		**0.266**		**0.566**	

Anti S-RBD IgG were measured at 1 (T1), 3 (T3), 6 (T6), 9 (T9) and 12 (T12) months following the second dose (D2) of vaccination and an additional assessment was planned one month after D3 (T1D3). Data on sex, age ≥65, vaccine switch at D3, off-treatment at vaccine, and IMiDs regimen are not shown, as no statistical significance was found at any time-point.

^a^Immunoparesis was defined by 2 uninvolved immunoglobulin class concentration below the lower limit.

^b^BNT162b2 (Pfizer-BioNTech) vaccine is comparator.

S-RBD, receptor binding domain (RBD) of the S1 subunit of SARS-CoV-2 spike (S) protein; Ig, immunoglobulins; M, male; PMN, polymorphonuclear leukocytes; D1, first vaccine dose; D2, second vaccine dose; D3, third vaccine dose; PIs, proteasome inhibitors; IMiDs, immunomodulatory drugs; anti-CD38 MoAbs, anti-CD38 monoclonal antibodies; ASCT, autologous stem cell transplantation; CR, complete response; VGPR, very good partial response; PR, partial response; SD, stable disease. Bold value represents statistical significance.

A multivariate analysis performed at T3 (e.g., before the booster dose) with a R^2^-adjusted of 0.4398, confirmed ≥CR (375.62, 95% CI 75.38-675.86, p=0.0149), receiving lenalidomide maintenance (552.82, 95% CI 78.36-1027.28, p=0.0230) and to have obtained a cellular response to the vaccine (526.94, 95% CI 211.39-842.49, p= 0.0014) as independent predictors of higher antibody titers (**Table 4**). Being in ≥CR (OR: 3.69, 95% CI 1.33-10.78, p=0.0138) and receiving lenalidomide maintenance (OR: 6.56, 95% CI 1.89-31.11, p=0.0067) were also related to the achievement of the highest median anti S-RBD IgG level (2500 U/mL) at 1 month after the booster dose (D3) (**Supplemental Table 2S**). Receiving lenalidomide maintenance (631.10, 95% CI 218.10-1044.03, p=0.0032) and having obtained a cellular response (514.10, 95% CI 116.89-911.23, p= 0.0118) significantly predicted for increased humoral response at T12 (overall R^2^-adjusted of 0.3381) (**Table 4**).

**Table 4:** Multivariate analysis of variables associated with humoral response at the different time-points.

	T3			T12^a^		
Variable	Estimate	95% CI	P-value	Estimate	95% CI	P-value
Anti-CD38 MoAbs regimen	-237.48	-643.05 -168.09	0.2472	–	–	–
PIs regimen	-206.13	-585.96 -173.71	0.2832	–	–	–
Lenalidomide maintenance	552.82	78.36 -1027.28	**0.0230**	631.10	218.10 -1044.04	**0.0032**
First line therapy	35.36	-317.76 - 388.49	0.8425	–	–	–
≥CR	375.62	75.38 -675.86	**0.0149**	280.30	-102.13 - 662.70	0.1489
Immunoparesis^b^	88.81	-289.45 - 467.07	0.6415	–	–	–
mRNA-1273 (D1+D2)^c^	181.40	-134.69 - 497.49	0.2567	370.30	-47.36 - 787.87	0.0815
Positive cellular response	526.94	211.39 - 842.49	**0.0014**	514.10	116.89 -911.23	**0.0118**

Multivariate analysis of variables related to humoral response was performed at 3 months (T3) and 12 months (T12) following the second dose of vaccination (D2).

^a^To construct the model, at T12 all variables found to be significant in the univariate analysis were considered, excluding non-significant coefficients, then performing model nested selection by comparing the adjusted R-squared (Complete: 0.2882, Nested: 0.3383).

^b^Immunoparesis was defined by 2 uninvolved immunoglobulin class concentration below the lower limit.

^C^BNT162b2 (Pfizer-BioNTech) vaccine is comparator.

CI, confidence interval; anti-CD38 MoAbs, anti-CD38 monoclonal antibodies; PIs, proteasome inhibitors; CR, complete response D1, first vaccine dose; D2, second vaccine dose. Bold value represents statistical significance.

### 3.3 Cellular immune response

Ninety-nine and 94 patients were assessable for CMI response at T3 and T12, respectively. In 5 patients in each of these two subgroups results of IGRA assay turned out to be not evaluable due to lymphopenia (≤0.5 x 10^9^/L), ultimately leading to available CMI response data in 94 patients at T3 and 89 patients at T12. Thirty-two out of 94 patients (36.2%) developed positive IGRA results at T3. Interestingly, all these patients were seropositive and had a significantly higher median anti S-RBD IgG level than those lacking cellular immunity (1264 U/mL [range 23.6-2500] vs 147 U/mL [0.4-2500], p<0.0001). Nine (9.6%) patients were double-negative for both humoral and cellular response.

At a median of 5 (IQR 4-6) months after the third dose (corresponding to T12), 42 (47.2%) patients displayed a positive IGRA response, and their median antibody titer was significantly higher compared with the subgroup showing a persistently negative cellular response (2500 U/mL [range 9.54-2500] vs 782 U/mL [range 0.4-2500], p=0.0001. Only 1 patient (1.1%) remained double negative at T12.

In univariate analysis, the following factors resulted positively related to the development of cellular response at both T3 and T12: response ≥CR, ongoing treatment with lenalidomide maintenance and prior ASCT (**Table 5**). In addition, having received the vaccine while on or soon after first-line therapy was associated with IGRA reactivity at T3. On the contrary, immunoparesis at all time-points and anti-MM therapies with PIs or anti-CD38 MoAbs at T12 were negative predictors of cellular response.

**Table 5:** Univariate analysis of variables associated with cellular response at the different time-points.

	T3			T12		
Variables	Frequency	Univariate analysis		Frequency	Univariate analysis	
	N reactive/evaluable pts (%)	OR (95% CI)	p-value	N reactive/evaluable pts (%)	OR (95% CI)	p-value
Sex M	48/94 (52.8%)	2.37 (0.93 - 6.34)	0.055	47/89 (52.8%)	1.16 (0.46 - 2.90)	0.832
Age ≥65	24/94 (53.9%)	1.52 (0.51 - 4.92)	0.468	23/89 (25.8%)	0.77 (0.26 - 2.20)	0.633
PMN ≤1.0 x 10 (9)/L	16/86 (18.6%)	1.08 (0.29 - 3.75)	1.000	18/82 (22.0%)	0.39 (0.09 - 1.34)	0.114
Immunoparesis^a^	57/86 (66.3%)	0.32 (0.11 - 0.89)	**0.019**	53/82 (64.6%)	0.26 (0.08 - 0.73)	**0.006**
mRNA-1273 (D1+D2)^b^	55/94 (58.5%)	2.23 (0.85 - 6.17)	0.085	52/89 (58.4%)	1.90 (0.75 - 4.97)	0.196
Off-treatment	7/94 (7.4%)	1.35 (0.19 - 8.55)	0.701	6/89 (6.7%)	6.10 (0.64 - 299.79)	0.096
Prior lines >1	38/94 (40.4%)	0.31 (0.10 - 0.85)	**0.016**	35/89 (39.3%)	0.51 (0.19 - 1.32)	0.136
PIs regimen	27/94 (28.7%)	0.40 (0.12 - 1.20)	0.098	22/89 (24.7%)	0.24 (0.062 - 0.79)	**0.013**
IMiDs regimen	69/94 (73.4%)	2.15 (0.71 - 7.41)	0.155	70/89 (78.7%)	0.99 (0.32 - 3.13)	1.000
IMiDs + PIs regimen	12/94 (12.8%)	0.55 (0.09 - 2.44)	0.526	14/89 (15.7%)	0.26 (0.04 - 1.07)	**0.044**
Anti-CD38 MoAbs regimen	28/94 (29.8%)	0.62 (0.20 - 1.74)	0.357	28/89 (31.5%)	0.25 (0.08 - 0.73)	**0.006**
Lenalidomide maintenance	33/94 (35.1%)	4.09 (1.54 - 11.33)	**0.003**	30/89 (33.7%)	6.75 (2.31 - 22.11)	**<0.001**
Prior ASCT	62/92 (67.4%)	5.61 (1.67 - 24.75)	**0.002**	56/87 (64.4%)	3.45 (1.26 - 10.19)	**0.013**
≥CR	47/94 (50.0%)	2.54 (0.99 - 6.76)	**0.053**	57/89 (64.0%)	3.46 (1.27 - 10.13)	**0.008**
≥VGPR	68/94 (72.3%)	3.09 (0.98 - 11.74)	**0.054**	73/89 (87.6%)	1.61 (0.47 - 5.99)	0.423
≥PR	82/94 (87.2%)	1.81 (0.41 - 11.20)	0.526	82/89 (92.1%)	2.36 (0.36 - 26.12)	0.439
≤SD	12/94 (12.8%)	0.55 (0.09 - 2.44)	0.526	7/89 (7.9%)	0.42 (0.04 - 2.77)	0.439

SARS-CoV-2 specific IFNγ T cell response by IGRA (Interferon-Gamma Release Assays) test was assessed at 3 months (T3) and 12 months (T12) following vaccination.

a Immunoparesis was defined by 2 uninvolved immunoglobulin (Ig) class concentration below the lower limit.

b BNT162b2 (Pfizer-BioNTech) vaccine is comparator.

N, number; pts, patients; OR, odds ratio; CI, confidence interval; M, male; PMN, polymorphonuclear leukocytes; D1, first vaccine dose; D2, second vaccine dose; PIs, proteasome inhibitors; IMiDs, immunomodulatory drugs; anti-CD38 MoAbs, anti-CD38 monoclonal antibodies; ASCT, autologous stem cell transplantation; CR, complete response; VGPR, very good partial response; PR, partial response; SD, stable disease. Bold value represents statistical significance.

Multivariate analysis identified receiving lenalidomide maintenance (OR: 3.18, 95% CI 1.01-0.3, p=0.0484) as the only independent predictor of positive cellular response at T3 (**Table 6**). At T12, being in response ≥CR (OR: 4.68, 95% CI 1.40-18.44, p=0.0172) was an independent factor favourable contributing to T-cell response, while regimens containing PIs (OR: 0.12, 95% CI 0.02-0.57, p=0.0130) or anti-CD38 MoAbs (OR: 0.15, CI 95% 0.02-0.72, p=0.0249) were associated with a diminished cellular response (**Table 6**).

**Table 6:** Multivariate analysis of variables related to cell-mediated immune response.

	T3			T12		
Variable	Odds ratio	95% CI	P-value	Odds ratio	95% CI	P-value
Anti-CD38 MoAbs regimen	/	/	/	0.15	0.02 - 0.72	**0.0249**
PIs regimen	/	/	/	0.12	0.02 - 0.57	**0.0130**
Lenalidomide maintenance	3.18	1.01 - 10.32	**0.0484**	1.31	0.24 - 6.91	0.7487
First line therapy	1.75	0.57 - 5.51	0.3301	/	/	/
≥CR	2.37	0.90 - 6.42	0.0832	4.68	1.40 - 18.44	**0.0172**
Immunoparesis	0.78	0.24 - 2.68	0.6790	0.75	0.20 - 2.90	0.6667

Multivariate analysis of variables related to cell-mediated immune response was performed at both assessment of IGRA (interferon gamma release assay) test at 3 months (T3) and 12 months (T12) following the second dose of vaccination.

CI, confidence interval; anti-CD38 MoAbs, anti-CD38 monoclonal antibodies; PIs, proteasome inhibitors; CR, complete response. Bold value represents statistical significance.

At last, ROC analysis revealed an anti S-RBD IgG level ≥346 U/mL as being the optimal cut-point associated with the development of a cellular response, with reasonable test sensitivity (94.1%), specificity (75.4%) and respective Youden index (0.695) (**Supplemental Figure 1S**). More in depth, the probability of a positive cellular response was 20 times higher in patients who achieved this cut-off (OR 20.6, 95% CI 6.89-78.01, p<0.0001).

### 3.4 Vaccine site reactions and breakthrough infections

Twenty-one MM patients experienced at least one adverse event (AE) related to the first two vaccine doses. All AEs reported were grade 1–2, transient, and mainly consisting in fatigue, mild temperature increase and pain in the injection site. No AE of grade 2, or higher, were reported following D3.

Documented SARS-CoV-2 infection occurred in 11 MM patients after D2 (n=2) or D3 (n=9). Among these, 2 patients were asymptomatic, 8 developed mild COVID-19 and 1 patient presented a severe illness requiring hospitalization. This patient had a prior history of advanced RRMM in progression at the time of infection and failed humoral and cellular immunogenicity after full vaccination. Six patients received antiviral medications (n=5) and monoclonal antibodies (n=1) to treat COVID-19. None of the infected patients died for COVID-19.

The main characteristics of patients who develop breakthrough infection are reported in **Supplemental Table 3S**.

## 4 Discussion

Several studies, mainly focusing on humoral data obtained a few weeks after the second dose, have described a highly variable, but impaired antibody response to anti-SARS-CoV-2 vaccination in patients with MM. However, the long-term antibodies kinetic and T-cellular response to vaccines, their protective activity against COVID-19 infection in MM and the real immunogenicity of the booster dose still remain to be elucidated.

To the best of our knowledge, this is the first prospective study which was specifically designed to assess the long-term kinetics of humoral and cellular responses to three doses of SARS-CoV-2 mRNA vaccines. For this purpose, anti S-RBD IgG titers were serially monitored every three months for up to 1 year after the second vaccine dose and for up to 6 months after the third dose in an observational cohort of 102 COVID-19-naive MM patients. Humoral response by Elecsys^®^ anti-SARS-CoV-2 ECLIA assay and T cell response by IFNγ-based IGRA assay were evaluated simultaneously at 3 and 12 months. Serological response of MM patients was compared with that of a control group of 63 health-care workers receiving two doses of anti-SARS-CoV-2 mRNA vaccines in the same period.

In this real-world study, the seroconversion rate after the first two doses of SARS-CoV-2 mRNA vaccines was 88.2% at T1 and increased up to 93% at T6, a value lower than in healthy controls, but one of the highest so far reported in similar studies. Heterogeneities among different series of patients in terms of their demographic, disease and treatment characteristics, may explain, at least in part, these discrepancies. In addition to the wide range of seroconversion failures, between 16% and 39%, the use of different assays and threshold levels to define a response to vaccination might have contributed to variable results reported by different groups (10–13). A slightly lower median age, less heavily pre-treated disease, less frequent exposure to daratumumab and lack of B-cell maturation antigen (BCMA)-targeted therapies were the main characteristics of our series of patients compared with those of other trials. All these factors might have contributed to the high seroconversion rate and to the persistence of anti S-RBD antibodies until 6 months after the second dose of vaccines. However, at this timepoint the median IgG titer was approximately 2-fold lower than at T1.

Notably, the third dose resulted in at least a 10-fold increase in the median antibody titer after 1 month from administration of vaccines. Antibody levels did not decline at a median of 5 months later and ultimately pushed the seropositivity rate in the subgroup of MM patients to that of the control group. Indeed, 99% of evaluable MM patients reached the seroconversion at T12, while a single patient with relapsed/refractory disease remained seronegative. The optimization of humoral response after the third dose observed in our study is consistent with results from other studies (17–19). In one report, 28% of patients remained seronegative after the second dose and 88% of these achieved sero-conversion after the third dose. Consistent with these results, our findings highlight the role of the booster dose to stimulate and renew vaccine immunogenicity, especially in immunocompromised patients, even if they did not achieve a detectable humoral response after the second dose.

In addition, we also aimed at sequentially assessing cellular immune response, a setting which was less commonly investigated in most of the studies reported so far (16–18, 22–25). For this purpose, we used the ELISPOT IFNγ release assay (IGRA), which is easier to use compared with other techniques (28, 29). Consistent with other studies (22, 30), after the first two doses CD4+ and CD8+ T-cell-mediated cytokine response was elicited in one third of patients and increased up to 47% after the booster dose (17, 18). Differently from reports by other groups (22, 24), all our patients exhibiting an IGRA-reactive test had obtained a humoral response; the development of a CMI response emerged as an important and independent predictor of increased median antibody levels, both before and after D3. After D2, 9.6% of our patients were double-negative for both humoral and cellular response and dropped down to 1% after the third dose.

Other studies reported an association between positive IGRA test and humoral response following the second dose of vaccination, suggesting the need for serological testing after vaccination to identify the subgroup of patients who fail to mount an optimal immunization (16, 23). These latter patients are at increased risk of infections and related complications, and might benefit from a revaccination strategy including sequential booster doses or prophylactic infusions of new anti-SARS-CoV-2 monoclonal antibodies adapted against the novel strains.

In addition, by using a ROC analysis, we showed that achieving an anti S-RBD IgG level ≥346 U/mL was related to a 20-fold enhanced probability of a positive cellular response, a finding that needs further confirmation by additional studies.

Additional end-points of our study included the correlation between immunogenicity, type of vaccine and patients’ characteristics. Consistent with other reports, immunoparesis and a sub-optimal hematological response were strong predictors of lower immunogenicity in univariate and multivariate analyses (10, 12, 15, 31). Immune response was enhanced in patients sparing steroids and receiving post-ASCT lenalidomide maintenance, a finding that supports the recommendation to avoid lenalidomide discontinuation before vaccination (5, 6, 10, 24, 32–34). By the opposite, and consistent with other reports, active therapies including PIs and anti-CD38 MoAbs were associated with reduced humoral and cellular responses, even after the third dose (9, 11, 13, 14, 35). Finally, the prolonged time lapse between ASCT and subsequent vaccination explained the lack of any negative impact of transplant on humoral and CMI response.

In our study, the overall SARS-CoV-2 breakthrough infection risk was consistent with that previously reported in the MM population and mainly characterized by mild symptoms due to the omicron variant after D3 (36). The low number of patients, their heterogeneity, in terms of disease status, ongoing therapies and vaccine immunogenicity did not allow to identify infection predictive factors.

The main strength of this study was the design aimed at evaluating at definite time-points humoral and CMI responses to three doses of COVID-19 mRNA vaccination and at monitoring the long-term kinetics of response in a relatively large series of MM patients in a real-world setting. The study has some limitations. The first is related to the anti-spike protein antibody assay, that might be less predictive of immune protection when compared with neutralizing antibodies titer, though more easily available and applicable in routine clinical practice (37–39). The IGRA test used to assess cellular immunity was impaired by severe lymphopenia, a finding which ultimately led to exclude 5 patients from the analysis at two different time points. In addition, cellular immunity was not evaluated in healthy controls. Lastly, we evaluated vaccine-induced immune response against a single strain of wild-type SARS-CoV-2 strain (wild-type), while results may have been different in the presence of variants of concern, such as Omicron.

In conclusion, results from this study support the benefit of the third dose of anti-SARS-CoV-2 mRNA vaccines in enhancing both humoral and cellular immune responses. In particular, the seroconversion rate was pushed up to 99% and a 10-fold increase in the median level of anti-S antibodies was detected, with persisting levels for up to 5 months after vaccination. T cell response was less efficiently optimized, though elicited by approximately 47% of patients. In addition, the booster dose was likely to promote immunogenicity renewal in patients failing a detectable humoral and cellular response after the second dose, a finding reflected by the reduced rate of double-negative patients from 9.6% to 1%. Immune response to vaccination was enhanced by deep hematological response and on-going treatment with lenalidomide, while it was impaired by PIs and anti-CD38 MoAbs. Additional studies are required to further explore the immunogenicity and durability of responses elicited by booster doses of vaccines sequentially given to MM patients. In addition, a more careful identification of severely immunocompromised patients at higher risk of developing severe disease and related complications is needed. For these patients, personalized approaches including prophylactic infusions of monoclonal antibodies or re-vaccination with variant-specific vaccines are required.

## Data availability statement

The raw data supporting the conclusions of this article will be made available by the authors, without undue reservation.

## Ethics statement

The study was reviewed and approved by the Ethics Committee of the National Institute for Infectious Diseases Lazzaro Spallanzani in Rome and subsequently by the Comitato Etico Area Vasta Emilia Centro (CE-AVEC)., Via Albertoni 15, Bologna (BO), Italy. All participants provided written informed consent to participate in this study.

## Author contributions

KM designed the research study, analyzed the data and wrote the paper. EZ designed the research study, analyzed the data, discussed the results and critically revised the paper. LP, SR, IR, PT, SG, EF, MU, MT, FB, MP CT, EB, MM and AS collected data and revised the paper. GN and BR helped in the management of patients. VS, AK, AP performed the statistical analysis. LG and ML performed all laboratory analysis. SB supported manuscript preparation and critically revised the paper. MC and TL contributed to study design and critically revised the paper. All authors contributed to the article and approved the submitted version.
