# Supplementary material for: Long term follow-up of humoral and cellular response to mRNA-based vaccines for SARS-CoV-2 in patients with active multiple myeloma
Source: Front Oncol. 2023 May 25;13:1208741. doi: 10.3389/fonc.2023.1208741 (PMC10249866; doi:10.3389/fonc.2023.1208741)

Supplementary Material

LONG TERM FOLLOW-UP OF HUMORAL AND CELLULAR RESPONSE TO mrna-BASED VACCINES FOR SARS-COV-2 IN PATIENTS WITH ACTIVE MULTIPLE MYELOMA

**Katia Mancuso^1,2^, Elena Zamagni^1,2*^, Vincenza Solli^1,2^, Liliana Gabrielli^3^, Marta Leone^4^, Lucia Pantani^1^, Serena Rocchi^1,2^, Ilaria Rizzello^1,2^, Paola Tacchetti^1^, Stefano Ghibellini^1,2^, Emanuele Favero^1,2^, Margherita Ursi^1,2^, Marco Talarico^1,2^, Simona Barbato^1,2^, Ajsi Kanapari^2^, Flavia Bigi^1,2^, Michele Puppi^1,2^, Carolina Terragna^1^, Enrica Borsi^1,2^, Marina Martello^1,2^, Andrea Poletti^1,2^, Alessandra Scatà^1^, Giuliana Nepoti^1^, Barbara Ruffini^1^, Tiziana Lazzarotto^3,4^, Michele Cavo^1,2^**

*** Correspondence:** Prof. Elena Zamagni: [e.zamagni@unibo.it](mailto:e.zamagni@unibo.it)

# Cellular evaluation of immune response

Cellular immunogenicity was evaluated with an IFNγ T cell response specific for SARS-CoV-2 using the Interferon-Gamma Release Assays (IGRA) test (QuantiFERON Human IFN-gamma SARS-CoV-2, Qiagen®). This is a qualitative assay that uses specialized blood collection tubes (Nil tube, Ag1 tube, Ag2 tube, and a Mitogen tube). The Ag1 tube contains CD4+ epitopes derived from the S1 subunit (RBD) of the S protein and the Ag2 tube contains CD4+ and CD8+ epitopes from the S1 and S2 subunits of the S protein. The plasma sample from the Mitogen tube serves as a positive IFN-γ control for each specimen tested. The Nil tube adjusts for background (e.g., elevated levels of circulating IFN-γ or presence of heterophilic antibodies). The tubes are shaken to mix antigen with the blood and should be incubated at 37°C ± 1°C as soon as possible, and within 16 hours after collection. After an incubation period of 16-24 hours, the tubes are centrifuged, the plasma is processed, and the amount of IFN-γ (IU/mL) is measured by enzyme-linked immunosorbent assay (ELISA). Elevated response was defined as at least 0.15 IU/mL higher than the background IU/mL value of the Nil tube (Table 1S).

# Supplementary Tables

## Table 1S. Interpretation of Interferon-γ release test results for SARS-CoV-2 infection.

| **Nil**  **(IU/mL)** | **Ag1 Antigen minus Nil (IU/mL)** | **Ag2 Antigen minus Nil (IU/mL)** | **Mitogen minus Nil**  **(IU/mL)^a^** | **QFN SARS result** | | **Report/Interpretation** | |  |
| --- | --- | --- | --- | --- | --- | --- | --- | --- |
| ≤8.0 | ≥0.15 and  ≥25% of Nil | Any | Any | Reactive | | SARS-CoV-2 response detected | |  |
|  | Any | ≥0.15 and  ≥25% of Nil |  |  |  |  |  |  |
|  | <0.15 or ≥0.15 and  <25% of Nil | <0.15 or ≥0.15 and  <25% of Nil | ≥0.50 | Non- Reactive | | SARS-CoV-2 response NOT detected | |  |
|  | <0.15 or ≥0.15 and  <25% of Nil | <0.15 or ≥0.15 and  <25% of Nil | <0.50 | Indeterminate | | SARS-CoV-2 response and Mitogen cannot be detected | |  |
| ≥8.0^b^ | Any | | | |  | |  | |

^a^Responses to the Mitogen positive control (and occasionally Ag Antigen) can be outside the range of the microplate reader. This has no impact on test results. Values >10 IU/mL are reported by the QFN SARS software as >10 IU/mL.

^b^In clinical studies, less than 0.25% of subjects had IFN-γ levels of >8.0 IU/mL for the Nil value.

## Table 2S. Multivariate analysis of variables related to the highest median anti s-RBD IgG level (2500 u/ml) at 1 month after the third dose.

| **Variable** | **Odds ratio** | **95% CI** | **P-value** |
| --- | --- | --- | --- |
| **≥CR** | 3.69 | 1.33 -10.78 | **0.0138** |
| **Lenalidomide maintenance** | 6.56 | 1.89 - 31.11 | **0.0067** |
| **mRNA-1273 (D3)^a^** | 2.82 | 0.89 - 9.28 | 0.0797 |

^a^BNT162b2 (Pfizer-BioNTech) vaccine is comparator.

Abbreviations: CI, confidence interval; CR, complete response; D3, third vaccine dose.

## Table 3S. Characteristics of patients developing Covid-19 after receiving at least one mRNA vaccine dose.

|  | **N=11** |
| --- | --- |
| **Age**, (range) years | 71 (53-81) |
| **Lines of therapy**, median (range) | 1 (1-4) |
| **Treatment type,** N  Anti-CD38 MoAbs + PIs  Anti-CD38 MoAbs + IMiDs  ASCT (<3 months)  Lenalidomide maintenance  Ixazomib maintenance  Bendamustine | 2  2  2  3  1  1 |
| **MM treatment response at infection**, N  CR  VGPR  PR  PD | 6  3  1  1 |
| **Time between D2 and SARS-CoV-2 infection**, median (range) months | 8 (7-10) |
| **Anti S-RBD IgG level prior infection**, median (range) U/mL | 1148 (59.2-2500) |
| **IGRA test prior SARS-CoV-2 infection**, N  Reactive  Non-reactive | 4  6 |

Abbreviations: anti-CD38 MoAbs, anti-CD38 monoclonal antibodies; PIs, proteasome inhibitors; IMiDs, immunomodulatory drugs; ASCT, autologous stem cell transplantation; CR, complete response; VGPR, very good partial response; PR, partial response; PD, progressive disease; D2, second vaccine dose; S-RBD, receptor binding domain (RBD) of the S1 subunit of SARS-CoV-2 spike (S) protein; Ig, immunoglobulins; IGRA, interferon gamma release assay.

# Supplementary Figures

## Figure 1 S. Optimal cut-off of anti-s-RBD IgG level associated to the development of cell-mediated immune response.


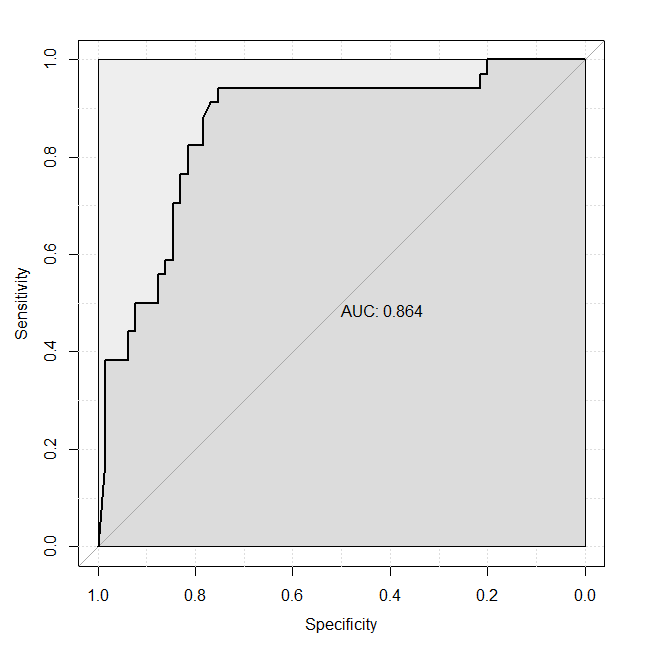

Supplement: Supplementary file 1 [file DataSheet_1.docx]
